# Electrochemical Valorization of Coconut Oil-Derived Fatty Acids: Toward a Sustainable Alternative for Fuel Additives

**DOI:** 10.1021/acsomega.5c08969

**Published:** 2026-02-26

**Authors:** Walber M. de O. Domingos, Thays L. Lemos, Jhudson G. L. Araujo, Elisama V. Dos Santos, Carlos A. Martínez-Huitle, Amanda D. Gondim, Lívia N. Cavalcanti

**Affiliations:** 28123 Federal University of Rio Grande Do Norte, Institute of Chemistry, Natal, Rio Grande Do Norte 59072-970, Brazil

## Abstract

The search for sustainable energy sources drives research in biomass conversion. This study investigates the electrochemical decarboxylation of fatty acids from coconut oil through the Non-Kolbe reaction, evaluating solvents, electrolytes, and applied voltage. Methanol achieved 100% conversion of substrate to product, while water and nonpolar solvents exhibited low reactivity. Inorganic bases like KOH, NaOH, and NaHCO_3_ were more effective than organic bases. High potentials (>10 V) favored oxygenated products, whereas moderate potentials (8–10 V) enhanced the production of linear α-olefins, such as 1-undecene and 1-tridecene. Results demonstrate that the electrosynthesis of fuel additives from fatty acids can offer a sustainable alternative to conventional thermochemical processes. The selective control of products via voltage and electrolyte/solvent choice presents a promising route for efficient and sustainable fuel additive production. This method requires lower energy input than conventional thermochemical routes, providing a more energy-efficient pathway for biomass valorization.

## Graphical abstract

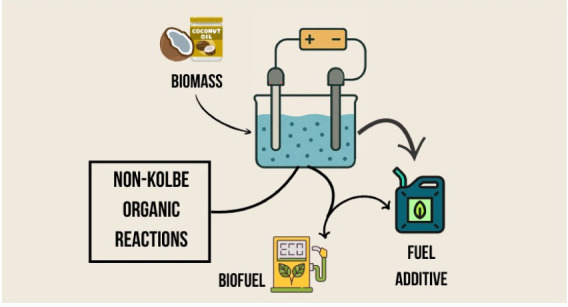

## 1 Introduction

The global energy demand, primarily driven by the intensive use of fossil fuels and the pursuit of carbon neutrality, has led to the search for low-carbon energy sources.^1–4^ In this context, biofuels from renewable biomass can provide a feasible alternative to traditional fuels. In order to employ these feedstocks as a sustainable energy source it is important to promote a deoxygenation of the biomass, a key step for upgrading organic acids and maximize their potential compatibility with existing engines and motors.^5–7^

There are various methods for biomass conversion into biofuels, including conventional approaches already implemented in the chemical industry, such as thermocatalysis. However, those traditional decarboxylation processes typically require harsh pressure and temperature conditions (above 200 °C and 200 bar).^8^ Over the years, the electrochemical conversion of bioderived compounds has gained increasing attention as no other alternative method currently exists that enables decarboxylation of fatty acids with such a high level of simplicity and sustainability.^9–12^ The industrial application of these biomass-derived products in organic electrosynthesis presents a promising alternative to reduce dependence on petroleum-based fuels.^13^

To afford renewable products through electrochemistry, the (Non-)­Kolbe reaction has been employed in early research for both traditional organic synthesis and macromolecule degradation.^6,8,13^ Several research groups have reported proposed reaction mechanisms, which involve multiple steps such as carboxylic acid deprotonation, alkyl radical generation, coupling, and CO_2_ disproportionation (Figure 1).^14^ Reaction parameters can significantly influence the outcome of an electrolysis process. In the pursuit of optimized reaction conditions, research efforts are primarily focused on parameters such as solvent, supporting electrolyte, temperature, voltage, current density, and faradaic efficiency, all of which directly influence the reaction mechanisms and product distribution.^15,16^

**Figure 1:**
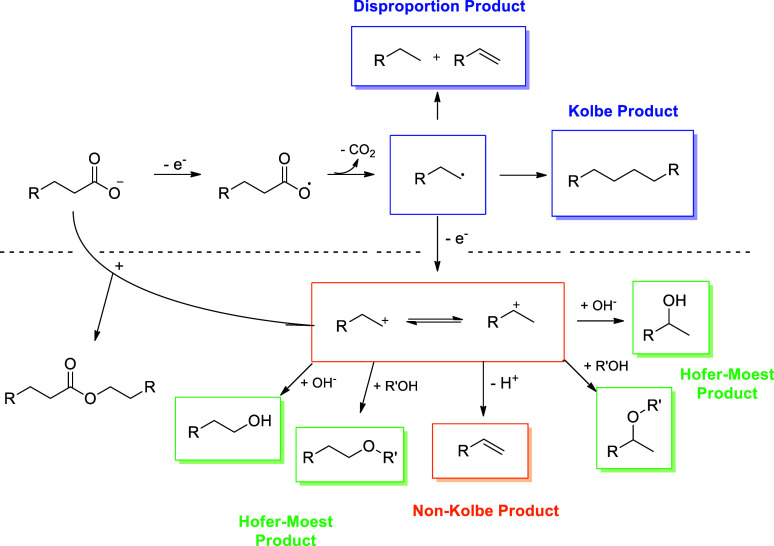
Scheme Electro-decarboxylation reaction (Non-)­Kolbe.

For example, a study on the effect of electrolytes in the Kolbe reaction of valeric acid using platinum as the anode, highlighted the advantages and disadvantages of adding supporting electrolytes for this process.^14^ In another study, the effect of the electrode was found determinant as employing platinum foil favored the Kolbe product at ≤4.0 V vs RHE and the Non-Kolbe product at 5.0 V vs RHE, whereas RuO_2_-TF (RuO_2_ thin film) promoted the Non-Kolbe product.^17^ The conversion of stearic and oleic acids (fatty acids present in rapeseed oil) was investigated on graphite anodes using methanol and ethanol as solvents to produce olefins and ethers as biofuel substitutes for fats and oils.^18^ The average selectivity for Non-Kolbe products exceeded 80%, while the formation of ethyl and methyl esters (byproducts) remained below 10%. The reported results show that electrochemical decarboxylation behavior of medium- to long-chain organic acids is crucial and requires further investigation.

Coconut oil holds significant relevance in the global chemical industry. Ranked seventh in worldwide production and industrial consumption, it is already employed in conventional biofuel synthesis.^5^ The oil can be efficiently obtained from coconut pulp extraction, yielding about 66 wt %, and is rich in lauric (C12:0) and myristic (C14:0) acids. These medium-chain fatty acids make it a compatible and promising feedstock for sustainable aviation fuel and other advanced biofuels.^19^

Conventional thermochemical approaches for fatty acid upgrading, such as hydrodeoxygenation and pyrolysis, typically demand high temperatures and hydrogen pressure, often yielding complex mixtures and consuming significant energy.^20^ Enzymatic strategies, while exceptionally selective, are hindered by scalability issues and the inherent fragility and cost of biocatalysts. In contrast, organic electrosynthesis replaces hazardous chemical reagents with a clean and tunable reagent: the electron.^21^ In this work, we demonstrate that electrochemical (Non-)­Kolbe decarboxylation enables the transformation of fatty acids derived from coconut oil into value-added products under mild conditions, offering an energy-efficient alternative to conventional biomass conversion routes.

This “green electron” paradigm enables reactions to proceed under mild and sustainable conditions, offering a controllable gateway to molecular complexity.^8,15,16,21^ The ability to modulate the applied potential in an electrochemical cell allows chemists to guide reactivity and selectivity with precision, minimizing side reactions while enhancing overall system efficiency. As a result, electrosynthetic methods have emerged as a powerful and environmentally conscious alternative for the valorization of fatty acids and related feedstocks.^22^

Reaction methodologies for obtaining mixtures of olefins and ethers from fatty acids are largely limited to the examples cited. Given that promising fatty acid profiles for biofuels and biobased products consist of triglycerides composed predominantly of C8 to C18 carbon chains (exclusively even-numbered substrates), investigating the electrochemical conversion of these fatty acids helps bridge existing knowledge gaps and assess the feasibility of this process. Herein we report a study of the influence of electrochemical parameters, such as solvent, electrolyte and voltage for the conversion of fatty acids derived from coconut oil into biofuels and aggregated values products to offer a potential technological alternative to the current biofuel production landscape.

## 2 Results and Discussion

The scarce studies on (Non-)­Kolbe-type organic electrosynthesis of vegetable oils prompted this study using coconut oil which is mainly composed of even-numbered fatty acids ranging from caprylic acid (C8:0) to stearic acid (C18:0) and oleic acid (C18:1). Following reported literature procedure, the biomass was hydrolyzed,^23^ and the relative fractions of fatty acid percentages were determined via GC-MS (Table 1).

**Table 1:** Composition of the Fatty Acid Mixture Obtained from the Hydrolysis of Coconut Oil

Fatty acids	Experimental results (%)^a^	%^b^
C8:0	1,2	9,2
C10:0	5,9	6,5
C12:0	47,1	45,6
C14:0	21,8	16,7
C16:0	11,8	8,2
C18:0	2,0	3,4
C18:1	10,2	6,5
C18:2	-	2,5

a Values determined by GC-MS using relative distribution.

b Reference values from Sabahannur e Alimuddin (2022).^39^

The fatty acid profile of coconut oil, based on GC-MS analysis, exhibits a carbon chain distribution consistent with literature data, although slight variations were observed between the obtained values and reference values. This discrepancy is expected, as the chemical composition of oils varies depending on cultivation methods, geographical origin, and postharvest processing technologies.^24^ Given its promising potential for electro-valorization, the mixture of fatty acids derived from coconut oil was used as a substrate in the electrosynthesis process.

Initially, the basic parameters for the electrolysis experiments were established. An undivided cell was chosen, as the investment costs for scaled-up processes are more favorable compared to divided cells with membranes, and potential loss across the diaphragm is avoided. The choice of a graphite electrode encompasses essential steps for reaction development, in addition to being a low-cost electrode.^25,26^

### 2.1 Solvent Effect

To investigate the influence of the solvent on the electrocatalytic oxidation via decarboxylation of fatty acids derived from coconut oil, various solvents—both polar and nonpolar—as well as solvent mixtures were selected.^24^ The reaction was initially conducted at low reagent and electrolyte concentrations. The choice of solvent as the first parameter aligns with the assertion that fine-tuning solvent selection is crucial, as solvents play an active role in determining the outcomes of electro-organic reactions.^27,28^

Water would be an environmentally ideal choice as a solvent due to its low cost and nontoxic nature.^29^ However, even a small fraction of long-chain carboxylates in water led to excessive foam formation, resulting from CO_2_ and H_2_ evolution at the anode and cathode, respectively. Consequently, the experimental results showed no product formation (Table 2, entries 2, 4, and 5).

**Table 2:**
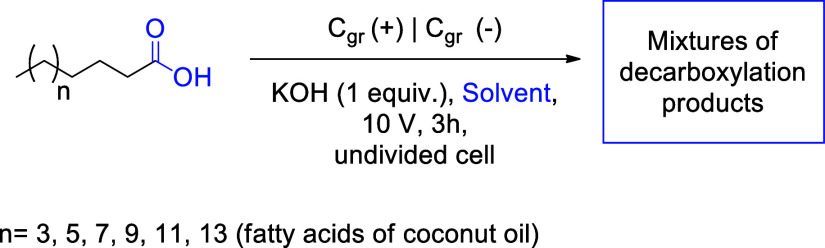
Study of Solvents for the Reaction Conditions of the Electro-Decarboxylation of Fatty Acids Derived from Coconut Oil^a^

Entries	Solvent	GC/MS Conversion (%)
1	MeOH	100
2	H_2_O	0
3	EtOH	94
4	MeOH:H_2_O (4:1)	Trace
5	MeOH:EtOH (1:1)	58
6	Acetone	Trace
7	MeCN	Trace
8	DCM	Trace
9	DMSO	0
10	THF	0
11	Toluene	0
12	DMA	0
13	iPrOH	0
14	Hexane	0
15	DMF	0
16	Et_2_O	0
17	Dioxane	0
18	CHCl_3_	0
19	EtOAc	0
20	NMP	0
21	(i-Pr)_2_O	0
22	Anisole	0

a Standard conditions: fatty acid (0.8 mmol), KOH (1 equiv), solvent (10 mL), r.t., constant cell potential (10 V). Conversion determined by relative distribution in GC-MS.

As shown in Table 2, among the listed solvents, methanol (entry 1) is considered the ideal solvent for Non-Kolbe electrolysis due to its high resistance to oxidation in these reaction systems. Additionally, other solvents were studied to explore the possibility of making the reaction methodology more attractive for biofuel production. For instance, ethanol (entry 3), a widely used biofuel in industry, could enable solvent incorporation into the final products for future applications.^30^

Since the initial conditions for these alcohol solvents yielded promising results, further optimization was pursued by analyzing the influence of electrolytes on the reaction. In the case of water, regarded as an ideal solvent due to its nontoxic nature, it would eliminate the need for subsequent separation processes between products and the electrolyte solution because of the low solubility of the products formed in aqueous medium.

However, in the experimental investigation (entry 2 in Table 2), it can be observed that water did not generate any compounds. During the course of the reaction, current passage and some reaction indicators, such as foam formation and temperature increase in the electrochemical cell, were noted. These reaction indications, combined with the fact that the substrate shows low solubility in water, support the findings reported by Ramos-Villaseñor, Sartillo-Piscil, and Frontana-Uribe (2024),^30^ who described the difficulty of obtaining favorable results in organic substrate–water solvent systems. On the other hand, although the conversion was slightly lower than under the initial conditions, the use of ethanol as solvent led to a high conversion rate, similar to that reported in the literature by Schröder and collaborators (2015).^18^

### 2.2 Effect Electrolyte/Solvent

This variable plays a crucial role in transforming the reaction medium, as the electrolyte/solvent ratio influences both electrical conductivity and interactions with the substrate. Therefore, both inorganic and organic basic electrolytes were evaluated. At this stage, conversion was observed under various electrolyte/solvent combinations; however, the most satisfactory yields were obtained when using inorganic basic electrolytes (Table 3).

**Table 3:**
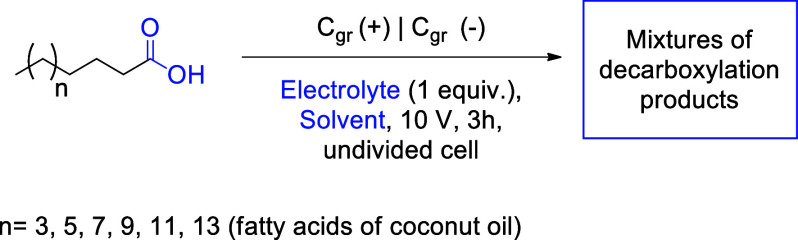
Study of the Electrolyte/Solvent Pair for the Electro-Decarboxylation Reaction Conditions of Coconut Oil-Derived Fatty Acids^a^

Entries	Electrolyte/Solvent	GC/MS Conversion (%)
23	NaOH/MeOH	100
24	NaHCO_3_/MeOH	100
25	K_2_CO_3_/MeOH	47
26	Et_3_N/MeOH	Trace
27	DIPEA/MeOH	0
28	Pyridine/MeOH	38
29	Et_2_NH/MeOH	Trace
30	NaOH/EtOH	20
31	NaHCO_3_/EtOH	14
32	K_2_CO_3_/EtOH	34
33	Et_3_N/EtOH	0
34	DIPEA/EtOH	0
35	Pyridine/EtOH	0
36	Et_2_NH/EtOH	0

a Standard conditions: fatty acid (0.8 mmol), electrolyte (1 equiv), solvent (10 mL), r.t., constant cell potential (10 V). Conversion determined by relative distribution in GC-MS.

Examining entries 23, 24, 25, 30, 31, and 34, all combinations of inorganic electrolytes with solvents exhibited positive conversion values. The notable influence of these electrolytes is attributed to monovalent alkali metal cations, which affect multiple electrode processes, particularly surface interactions, and overall efficiency. Supporting this idea, Ashraf, Mei, and Mul (2024) explored the electrochemical oxidation of acetic acid and demonstrated the significant impact of monovalent alkali metal cations on reaction efficiency.^32^

Furthermore, these findings confirm the crucial role of monovalent alkali cations in electrochemical decarboxylation, reinforcing the rationale for selecting inorganic bases for conversion.^33^ Zhang et al. (2024), while studying the effect of alkali ions on the conversion of octanoic acid into Kolbe products using platinum electrodes, also observed a similar pattern of ion influence on product conversion.^34^

Among the organic electrolytes, only one condition-pyridine (entry 28)-showed a conversion rate below 40%, while the others either exhibited no conversion or yielded unsatisfactory results. This is due to the lack of ion production from dissociation, which is essential for electrochemical decarboxylation reactions that occur at the electrode surface. In the presence of organic electrolytes, low electrical conductivity prevents effective conversion. Given the prominent conversion observed for the electrolyte/solvent pairs in entries 23 and 24, further optimization was pursued to evaluate the influence of these parameters in relation to voltage variation.

### 2.3 Effect of Voltage

As shown in Table 4, the effect of cell voltage was investigated. Experiments were conducted under constant potential, varying the applied voltage while keeping other reaction conditions constant: 0.8 mmol of fatty acid, 1 equiv of electrolyte, 10 mL of solvent, room temperature, and a reaction time of 3 h.

**Table 4:**
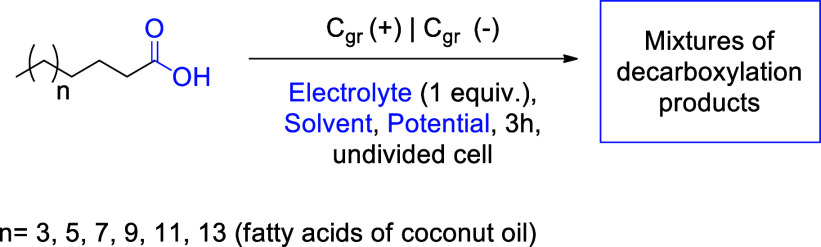
Study of the Potential for the Electro-Decarboxylation Reaction Conditions of Coconut Oil-Derived Fatty Acids^a^

Entries	Electrolyte/Solvent	Potential	GC/MS Conversion (%)
37	KOH/MeOH	15 V	100
38	NaOH/MeOH	15 V	100
39	NaHCO_3_/MeOH	15 V	100
40	KOH/EtOH	15 V	100
41	KOH/MeOH	8 V	100
42	NaOH/MeOH	8 V	100
43	NaHCO_3_/MeOH	8 V	100
44	KOH/EtOH	8 V	100
45	KOH/MeOH	6 V	100
46	NaOH/MeOH	6 V	100
47	NaHCO_3_/MeOH	6 V	100
48	KOH/EtOH	6 V	45
49	KOH/MeOH	4 V	96
50	NaOH/MeOH	4 V	Trace
51	NaHCO_3_/MeOH	4 V	89
52	KOH/EtOH	4 V	0

a Standard conditions: fatty acid (0.8 mmol), electrolyte (1 equiv), solvent (10 mL), r.t., constant cell potential. Conversion determined by relative distribution in GC-MS.

At 15 V, a water bath was used to maintain room temperature, as without it, the temperature exceeded 45 °C.^35^ With the increase in cell voltage, product conversion followed the same pattern observed with previous parameters, like the results at 8 V. However, at 6 and 4 V (entries 49 to 52), there were slight decreases in some cases and drastic reductions in others. Understanding these variations in conversion patterns is essential for determining the most effective conditions, as they directly influence the formation of valuable fuel additives, such as linear α-olefins and fatty alcohols.^31,36,37^

Analysis of the graphs reveals a clear shift in product distribution as the applied voltage varies. In Figure 2 (10 V), the formation of alcohol and olefins is predominant, while esters are notably present (10%) when ethanol is used as the solvent. As the voltage increases to 15 V (Figure 3), ester formation disappears in ethanol, suggesting that higher voltages favor simpler products, such as alcohols and olefins, possibly due to an increased rate of intermediate chain cleavage. However, at potentials above 10 V in methanol, the trend reverses, with oxygenated products, particularly fatty alcohols, becoming predominant.

**Figure 2:**
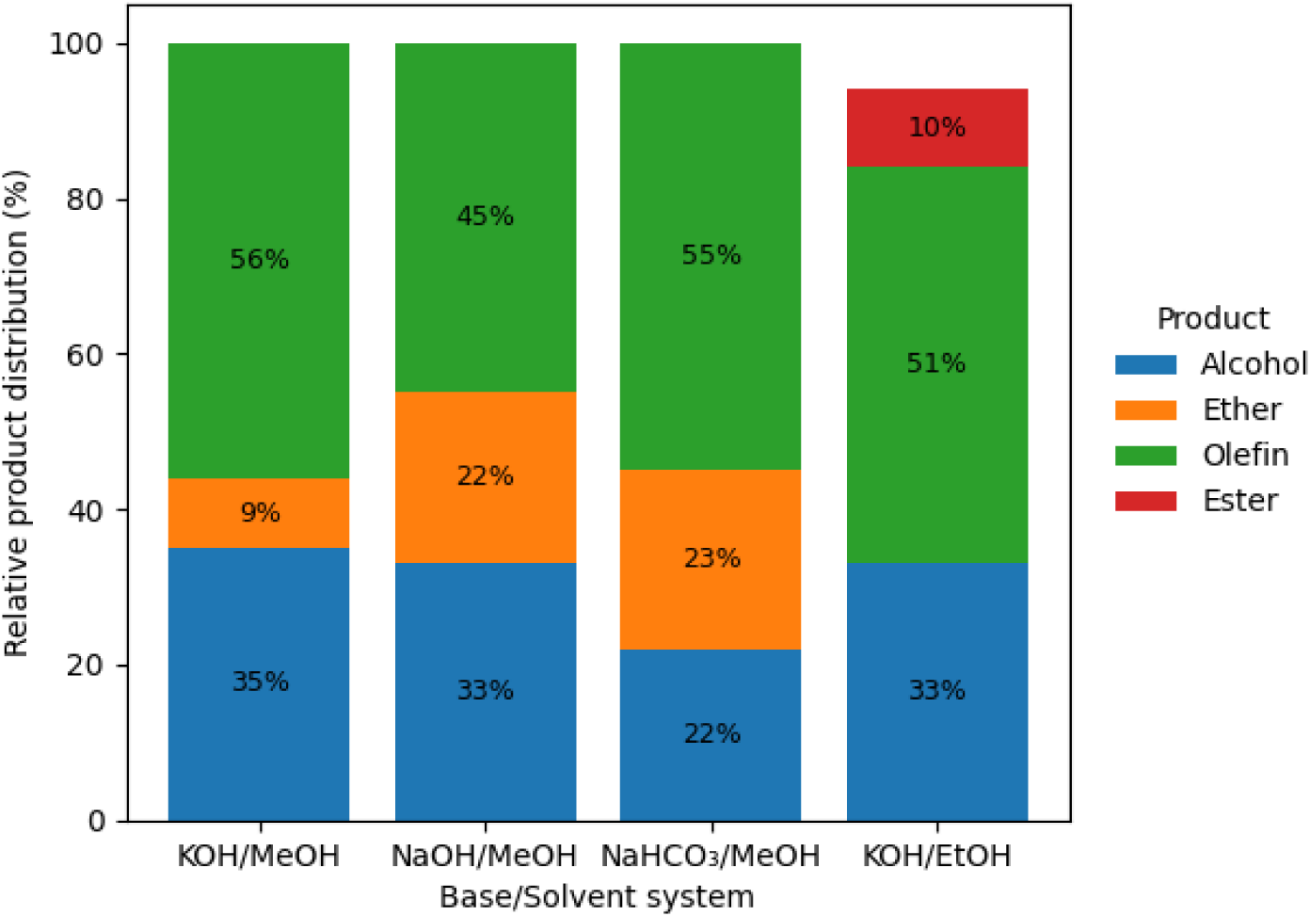
GC-MS-determined product distribution for the electro-decarboxylation of fatty acids at 10 V under constant potential, highlighting the effect of the base/solvent system on selectivity.

**Figure 3:**
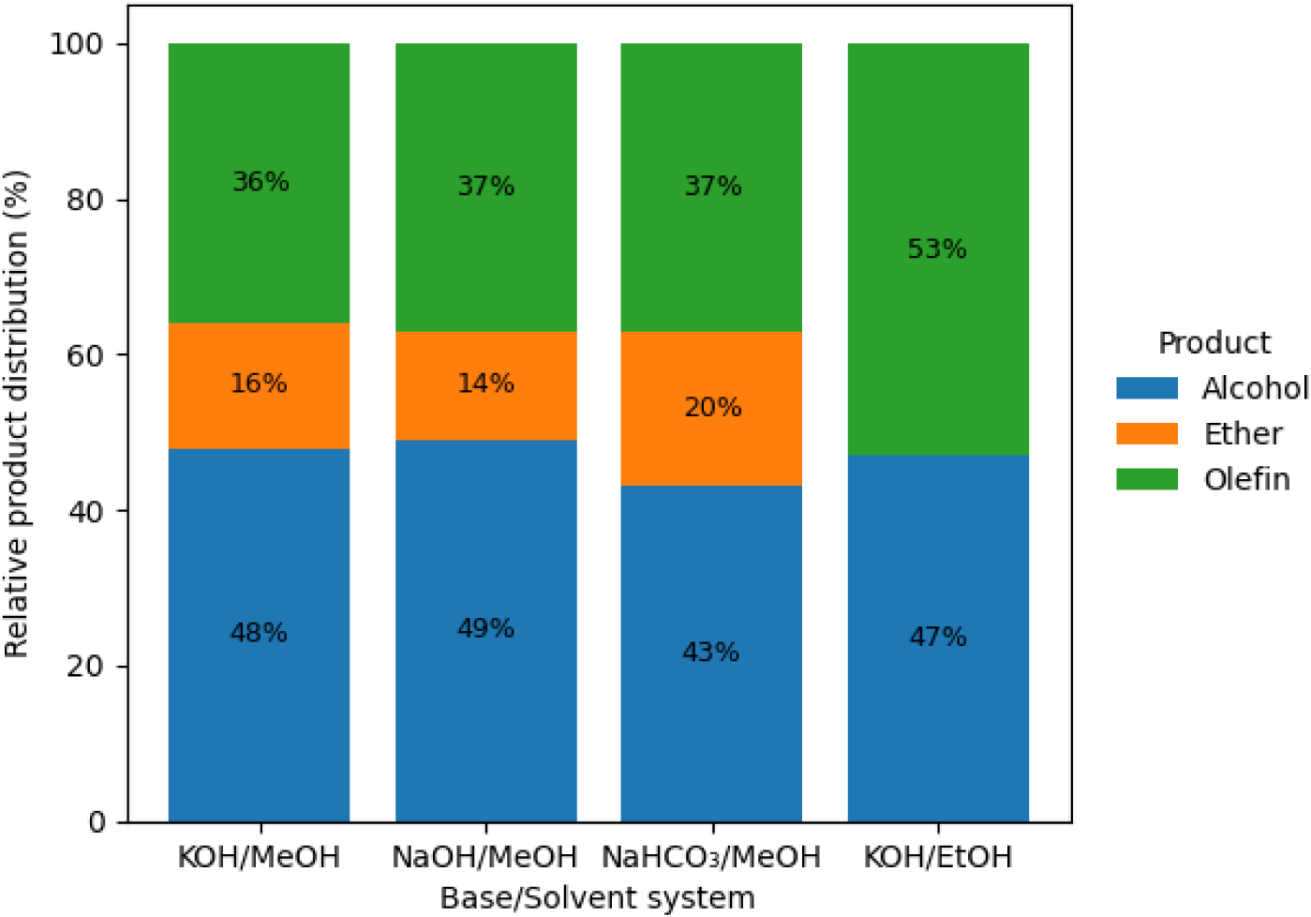
GC-MS-determined product distribution for the electro-decarboxylation of fatty acids at 15 V under constant potential, highlighting the effect of the base/solvent system on selectivity.

The product distribution analysis shown in Figure 4 reveals an interesting balance between energy efficiency and chemical conversion, particularly with NaHCO_3_/MeOH. Under these conditions, a decrease in potential leads to a 4% increase in olefin conversion. This makes the use of a moderate voltage more attractive, as it not only reduces energy costs but also enhances the yield of hydrocarbons, particularly linear α-olefins such as 1-undecene and 1-tridecene. In contrast, other methanol-based conditions favor the formation of oxygenated products.

**Figure 4:**
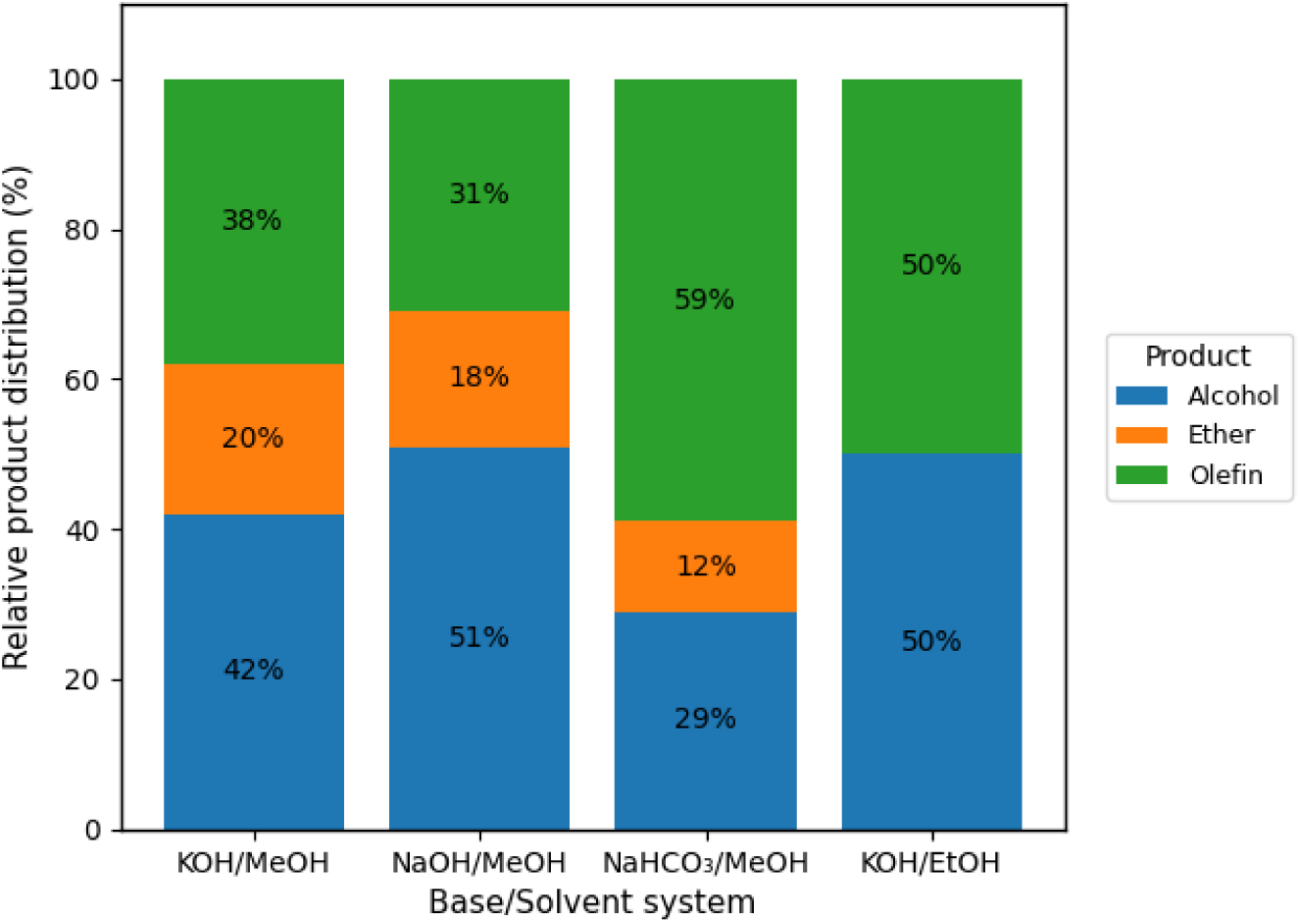
GC-MS-determined product distribution for the electro-decarboxylation of fatty acids at 8 V under constant potential, highlighting the effect of the base/solvent system on selectivity.

As the voltage is reduced to 6 V (Figure 5), product conversion remains proportional to the decrease in potential, alongside substrate interactions with the electrode surface and the influence of monovalent ions. Notably, under these conditions, when ethanol is used as the solvent, conversion is partial, yielding only alkenes. The decline in ether formation also suggests that lower voltages reduce secondary elimination and rearrangement reactions. In contrast, in Figure 6, applying a lower potential result in significantly reduced yields or even the inhibition of product formation.

**Figure 5:**
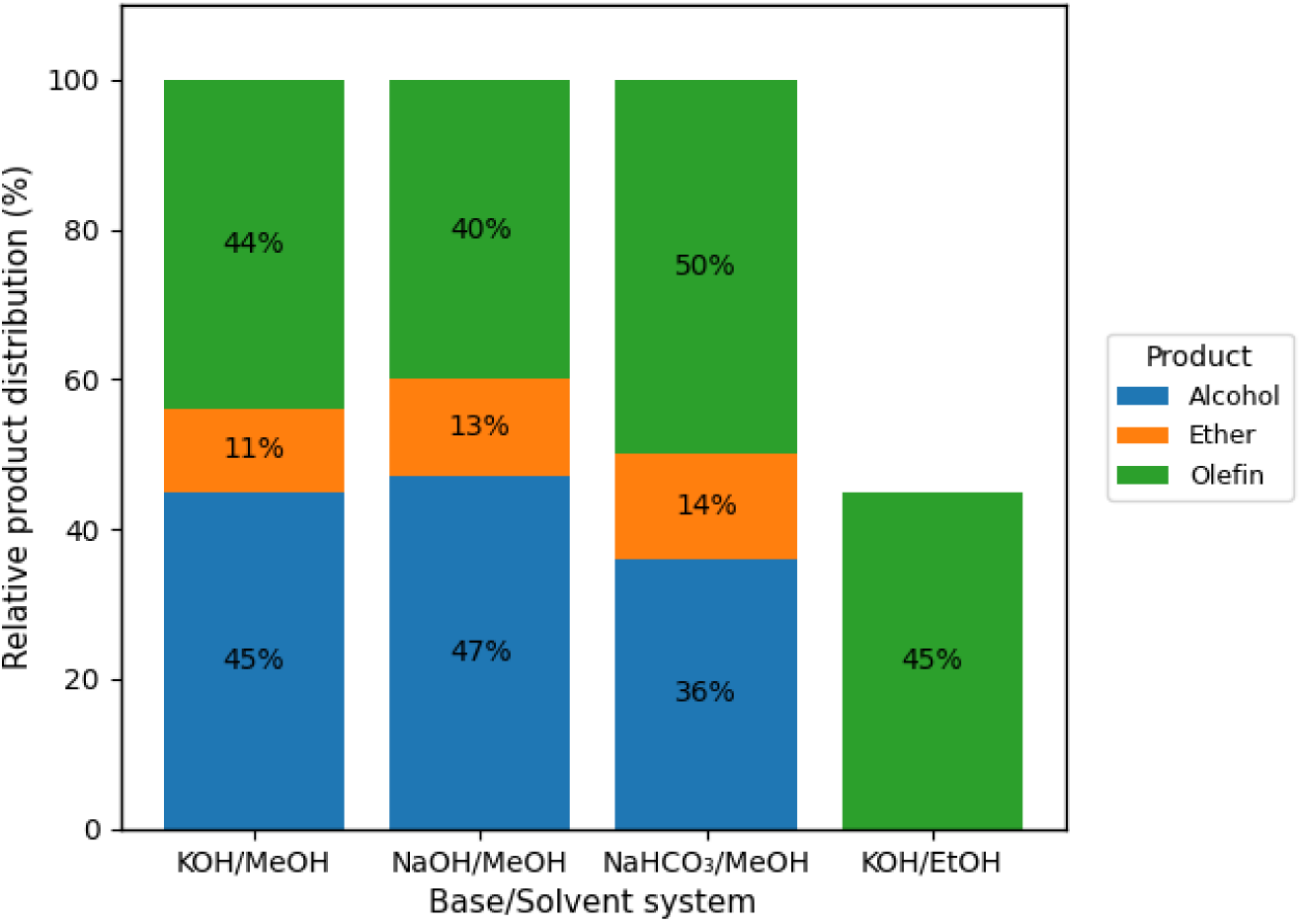
GC-MS-determined product distribution for the electro-decarboxylation of fatty acids at 6 V under constant potential, highlighting the effect of the base/solvent system on selectivity.

**Figure 6:**
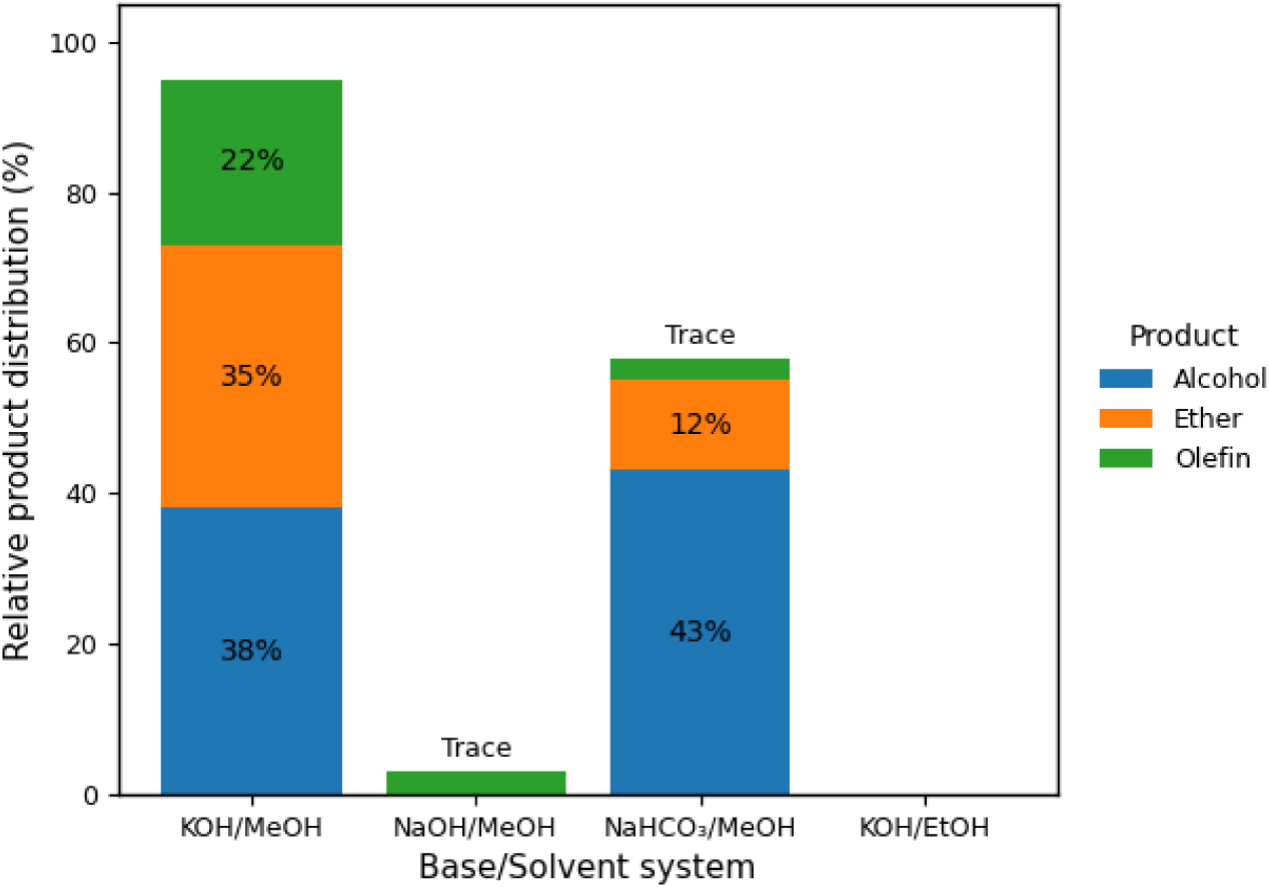
GC-MS-determined product distribution for the electro-decarboxylation of fatty acids at 4 V under constant potential, highlighting the effect of the base/solvent system on selectivity.

Thus, the implications of these results extend beyond the laboratory scope, offering practical contributions to industrial processes. Voltage control, combined with the strategic selection of electrolytes and solvents, enables the customization of reaction pathways to maximize the production of desired compounds. This approach provides advantages in both energy efficiency and electrochemical conversion, making Non-Kolbe electrolysis a versatile and promising tool for chemical applications.

Future investigations into the interactions between parameters, such as reaction time and reagent concentration, could provide a more comprehensive understanding of the process. Although technological challenges remain, this method presents an interesting and potentially competitive alternative in terms of energy efficiency compared to existing fuel additive production and hydrotreatment routes.

After multiple runs, a gradual degradation of the carbon electrode was observed, evidenced by a loss of surface conductivity and morphological changes visible and reflected in the variability of some experimental replications. This deterioration is likely associated with progressive surface oxidation promoted by the harsh anodic conditions and the formation of reactive intermediate species during the electrochemical process. Although carbon is generally robust and widely used in electrosynthetic reactions, repeated cycling can lead to the accumulation of adsorbed byproducts and the development of microfractures that gradually reduce electrode efficiency. Therefore, implementing a cleaning protocol or periodic electrode replacement may be necessary to ensure reproducibility across successive experiments.

#### 2.3.1 Variability and Product Distribution in Non-Kolbe Electrolysis

Under the evaluated conditions, the Non-Kolbe reactions exhibited a remarkably sensitive behavior toward both the nature of the solvent and the applied potential control. While the oxygenated products, mainly alcohols and ethers, emerged as evidence of the involvement of partially stabilized radical intermediates, the nonoxygenated products followed the classical C–C coupling pathway, with alkenes being the dominant outcome.

The variability among experimental runs, though limited, reflects the delicate competition between radical oxidation pathways and recombination processes. The standard deviation observed in the distribution of oxygenated products typically ranged between 3–7%, an interval that reveals subtle fluctuations in the reactivity of electrode-adsorbed species. For nonoxygenated products, the deviation tended to be smaller, approximately 2–4%, suggesting that the radical dimerization route (analogous to the classical Kolbe process) is more reproducible under identical electrochemical conditions.

This seemingly modest difference is, in fact, revealing. It indicates that Non-Kolbe reactions, though governed by well-described mechanisms, remain sensitive to subtle parameters such as the electrode surface state or the residual water content in the solvent. Minor variations in the oxidation potential or electrolyte purity can shift the balance between oxygenated and deoxygenated pathways, making the standard deviation an equally informative metric as the relative substrate-to-product conversion distribution.

### 2.4 Electrochemical Behavior of the System

The electrochemical behavior of fatty acid derivatives derived from coconut oil was investigated to rationalize the observed product distribution under different base and solvent systems. Rather than treating product formation as an empirical outcome, the results can be understood by considering a small number of fundamental electrochemical events that govern the fate of anodically generated intermediates.

Under basic conditions, fatty acids are present predominantly as carboxylate anions. Upon anodic polarization, these species undergo single-electron oxidation to generate carboxyl radicals, which rapidly decarboxylate to form alkyl radicals. This initial step is common to both Kolbe and Non-Kolbe pathways and represents the key electrochemical activation event.

In systems where olefin formation predominates, the reaction proceeds mainly through Non-Kolbe pathways. After decarboxylation, the alkyl radical undergoes further oxidation to a carbocationic species or radical cation, which subsequently eliminates a proton to form an olefin.

This pathway is favored under conditions that stabilize charged intermediates, such as polar protic solvents and bases that promote strong ion pairing at the electrode surface. The high olefin selectivity observed with bicarbonate-containing systems suggests that weaker bases limit rapid radical–radical coupling, allowing further oxidation to compete effectively. Thus, olefin formation can be viewed as the thermodynamically driven outcome of sequential electron transfer and proton loss, rather than a direct radical coupling event.

Alcohol formation arises from the interception of reactive intermediates by the solvent. Once alkyl radicals or carbocationic species are generated near the electrode surface, nucleophilic trapping by methanol or ethanol becomes competitive.

In this context, the solvent plays a dual role: it acts both as the reaction medium and as a reactant. The predominance of alcohol products in strongly basic methanolic systems is consistent with rapid solvent capture occurring faster than radical recombination or elimination processes. This mechanism explains why alcohol formation is particularly sensitive to solvent identity and base strength, while being less dependent on the intrinsic structure of the fatty acid chain.

Ether products are formed through pathways involving radical coupling with solvent-derived species. One plausible mechanism involves hydrogen abstraction from the solvent by alkyl radicals, generating solvent radicals that recombine with carbon-centered radicals to form C–O bonds.

Alternatively, ether formation may proceed through carbocationic intermediates followed by nucleophilic substitution by the alcohol solvent. In either case, ether formation reflects a balance between radical persistence and solvent participation.

The relatively lower abundance of ether products compared to alcohols suggests that these pathways are secondary and become significant only under conditions where radical lifetimes are sufficiently long.

Taken together, the electrochemical reaction network can be simplified into a unified mechanistic framework: anodic decarboxylation generates a common radical intermediate, whose fate is determined by the competition between radical coupling, further oxidation, and solvent trapping.

Rather than invoking multiple unrelated mechanisms, the observed product distribution emerges naturally from the interplay between electron transfer kinetics and chemical reactivity at the electrode–solution interface. Small changes in base strength or solvent identity therefore result in large shifts in selectivity by biasing this competition.

This perspective underscores the utility of electrochemical methods not merely as synthetic tools, but as platforms for controlling reaction pathways through precise modulation of the reaction environment.

### 2.5 Stability of the Reaction Products

In addition to formation pathways, the observed product distribution is strongly influenced by the relative stability of the products under the electrochemical conditions. Olefins, once formed, are comparatively stable toward further oxidation in the potential window employed, allowing them to accumulate in solution. Their lack of heteroatoms also limits secondary electrochemical or chemical transformations.

Alcohols exhibit high stability under basic conditions, particularly in alcoholic solvents, where they are thermodynamically favored and kinetically resistant to further oxidation at moderate anodic potentials. This stability contributes to their predominance in systems where solvent trapping is efficient. In contrast, ether products are less abundant, which may reflect their lower stability toward cleavage or oxidation, as well as their formation through secondary pathways that require longer radical lifetimes.

Therefore, the final product distribution reflects not only the kinetics of electrochemical generation but also the differential stability of each product class under the reaction conditions. This distinction is essential for understanding why certain products dominate even when multiple formation pathways are accessible.

During the electrochemical reaction, gas evolution is observed as an inherent consequence of the anodic decarboxylation process. At the anode, carboxylate species undergo single-electron oxidation, leading to the release of CO_2_ concomitantly with the formation of carbon-centered radical intermediates. This gas evolution is therefore directly associated with the primary reaction pathway and does not indicate undesired side reactions. At the cathode, minor hydrogen evolution may occur due to proton reduction, especially in protic solvents; however, under the applied electrochemical conditions, this process remains limited and does not significantly compete with the desired transformation. No evidence for the formation of other gaseous hydrocarbons was detected, suggesting that radical recombination pathways leading to volatile products are disfavored in this system. Overall, gas evolution is consistent with the proposed reaction mechanism and does not adversely affect product stability or selectivity.

### 2.6 Faradaic Efficiencies

The Faradaic efficiencies (FE) reflected the same delicate balance observed in product selectivity. Under optimized conditions, oxygenated products accounted for ca. 35–40% FE, whereas hydrocarbon-type (nonoxygenated) products consistently reached 55–60%, with the remainder attributed to minor side processes and background current. Such values proved reproducible within ±3% across independent trials.

Overall, the Faradaic data complement the product distribution trends, reinforcing the notion that *Non-Kolbe* electrolysis operates under a finely tuned interplay between radical lifetime and surface dynamics, where even small shifts in electron transfer efficiency can translate into measurable changes in selectivity.

Notably, our observed Faradaic efficiencies for nonoxygenated products and oxygenated products mirror the high selectivity approach reported by Zhang et al., who achieved ∼95% FE for Non-Kolbe products under aqueous conditions.^38,39^ Moreover, the comparatively lower reproducibility (higher standard deviation) in the oxygenated-product channel in our study aligns with the mechanistic insight of surface–adsorbate sensitivity discussed in their work, reinforcing the notion that subtle interfacial perturbations similarly govern selectivity in both systems.

### 2.7 Context within Existing Fatty Acid Upgrading Technologies

Although the present study demonstrates the feasibility of a mild electrochemical upgrading route at laboratory scale, it is important to note that industrial processing of fatty acids typically relies on hydrodeoxygenation and catalytic cracking, which operate under high temperature and pressure. Compared with these, the Non-Kolbe pathway provides a milder and potentially modular alternative, though current efficiencies and scalability remain under investigation.

While a full life-cycle or energy balance assessment is beyond the scope of this work, preliminary considerations can already be drawn from the intrinsic features of the electrochemical approach. The Non-Kolbe pathway operates under ambient pressure and relatively mild temperatures, avoiding the intensive energy input and hydrogen consumption typical of catalytic hydrodeoxygenation or cracking processes. Moreover, the modular nature of electrolysis allows direct coupling with renewable electricity sources, which could substantially reduce the overall carbon footprint if implemented on a larger scale.

Coconut oil represents an abundant and renewable lipid source in tropical regions; however, its deployment for energy applications must be weighed against its role in the food supply chain. Future iterations of this strategy would benefit from incorporating nonedible or waste-derived lipid feedstocks to enhance sustainability and minimize resource competition.

## 3 Conclusion

The present study demonstrated that the electro-decarboxylation of fatty acids derived from coconut oil can be optimized through the strategic selection of solvents, electrolytes, and applied voltage. Methanol, in combination with inorganic bases such as KOH and NaHCO_3_, resulted in the highest conversions to desirable products. Furthermore, voltage variation revealed significant control over product distribution, favoring the formation of olefins at moderate voltage and oxygenated compounds at lower voltage.

The results suggest that this approach could serve as a sustainable alternative to conventional processes based on high temperatures and pressures, contributing to the reduction of fossil fuel dependence. The electrosynthesis of biofuels from coconut biomass represents a promising advancement in the field of green chemistry and paves the way for future investigations into the scalability and industrial feasibility of this technology.

Finally, the electro-decarboxylation strategy demonstrated here could be expanded to larger-scale electrosynthetic platforms through the use of flow cells or modular electrochemical reactors. Such setups would allow for improved control of mass transport, heat management, and electrode stability during extended operation. Moreover, coupling this process with renewable electricity sources, such as solar or wind power, could greatly enhance its sustainability and reduce the overall carbon footprint. Continued studies on electrode durability and cell design will be key to translating these laboratory findings into practical large-scale applications.

## 4 Experimental Section

### 4.1 General Procedure for the Methodology of Studying the Electro-Decarboxylation of Fatty Acids

The methodology for analyzing the influence of parameters on the electro-decarboxylation of fatty acids was guided by the product distribution yield determined via GC-MS. The solvent and electrolyte variables were evaluated based on their optimal relative distributions.

The reactions were prepared according to the following procedure: In a clean electrochemical cell, dried in an oven and equipped with a magnetic stirring bar, fatty acids derived from coconut oil (177 mg, 0.8 mmol, 1 equiv), 10 mL of solvent, and an electrolyte (0.8 mmol, 1 equiv) were added. The reaction was maintained at room temperature under stirring until the base was completely dissolved. The cell was then sealed with a polyethylene holder containing copper filaments connected to graphite electrodes, which were linked via alligator clips to a DC power supply. A constant potential was applied for 3 h at room temperature.

For reaction product analysis, a 1 mL aliquot was taken from the reaction mixture and transferred to a borosilicate vial, followed by the addition of 1.0 mL of a 1.0 mol/L hydrochloric acid solution and 2 mL of hexane. After phase separation, a 200 μL aliquot of the hexane fraction was withdrawn and transferred to another vial, where 1.8 mL of UV/HPLC-grade *n*-hexane was added. Anhydrous sodium sulfate was then introduced as a drying agent. The sample prepared was subsequently submitted for qualitative analysis by GC-MS. The chromatograms were used to determine the relative distribution fractions of each product as well as the conversion efficiency.

### 4.2 Gas Chromatography–Mass Spectrometry (GC-MS)

The relative yields were obtained using a Shimadzu gas chromatograph (GC-2010) coupled to a mass spectrometry detector (GCMS-QP2020). The instrument was equipped with an RTX-5MS column, packed with 5% diphenyl and 95% dimethylpolysiloxane, with dimensions of 30 m in length, 0.25 mm in internal diameter, and 0.25 μm in film thickness.

Samples were prepared in UV/HPLC-grade hexane and injected at a temperature of 230 °C with an injection flow rate of 1 mL/min, using ultrahigh purity helium as the carrier gas. The chromatographic separation was performed using a temperature program that began at 40 °C, held for 4 min, followed by a heating ramp of 4 °C/min up to 180 °C, where it was maintained for 5 min. The temperature was then increased at a rate of 7 °C/min up to 270 °C and held for 3 min.

## Supplementary Material



## Notes

Detailed hydrolysis procedure; complete experimental description; electrochemical setup and configuration; list of materials, reagents, and solvents used (PDF)
