# Supplementary material for: Electrochemical Valorization of Coconut Oil-Derived Fatty Acids: Toward a Sustainable Alternative for Fuel Additives
Source: ACS Omega. 2026 Feb 26;11(9):14392–401. doi: 10.1021/acsomega.5c08969 (PMC12980160; doi:10.1021/acsomega.5c08969)
Supplement: Supplementary file 1 [file ao5c08969_si_001.pdf]

Electrochemical Valorization of Coconut Oil-Derived Fatty Acids: Toward a Sustainable Alternative for Fuel Additives

Walber M. de O. Domingos[a], Thays L. Lemos[a], Jhudson G. L. Araujo[a], Elisama V. dos Santos[a], Carlos A. Martínez-Huitle[a], Amanda D. Gondim[a], Lívia N. Cavalcanti[a*]

[a] *Federal University of Rio Grande do Norte, Institute of Chemistry, 59072-970, Natal, RN, Brazil*

* Corresponding author. Tel.: +55 (84) 3342-2323 ; e-mail: livia.cavalcanti@ufrn.br .

Supporting Information

Electrochemical Valorization of Coconut Oil-Derived Fatty Acids: Toward a Sustainable Alternative for Fuel Additives

Walber M. de O. Domingos[a], Thays L. Lemos[a], Jhudson G. L. Araujo[a], Elisama V. dos Santos[a], Carlos A. Martínez-Huitle[a], Amanda D. Gondim[a], Lívia N. Cavalcanti[a*]

[a] *Federal University of Rio Grande do Norte, Institute of Chemistry, 59072-970, Natal, RN, Brazil*
* Corresponding author. Tel.: +55 (84) 3342-2323 ; e-mail: livia.cavalcanti@ufrn.br .

Contents

1. General Procedure for Coconut Oil Hydrolisis S3
2. General Procedure for Electrodescarboxylation S4
3. Gas Chromatography Coupled with Mass Spectrometry (GC-MS) S6
4. Materials S6
4.1 Solvents S6
4.2 Reagents S6
5 References S6

1 . General Procedure for Coconut Oil Hydrolysis

The free fatty acids were obtained through experimental adaptation of the procedure established in the literature [1].

In a beaker, 5.0 g of coconut oil were measured, and in a volumetric flask, a saponifying solution of 50 mL of ethanolic KOH (90%, 1.75M) was prepared. After measuring the oil mass and preparing the solution, 30 mL of the solution and 5.0 g of the oil were mixed in a round-bottom flask and heated to 65 °C under constant stirring until the starting material was completely consumed. The reaction was monitored by thin-layer chromatography. At the end of the process (3 hours), the reaction was transferred to a beaker, and 30 mL of water were added to the mixture. The unsaponifiable matter was separated by hexane extraction (adding 20 mL, twice). The alcoholic phase containing the soaps was acidified to pH 1 with hydrochloric acid (6 M), and the fatty acids were recovered by hexane extraction. The extract was washed with distilled water until the pH reached neutrality. The hexane fraction was dried with sodium sulfate, and the solvent was rotary-evaporated to obtain 4.76 g of fatty acids (Figure S1).

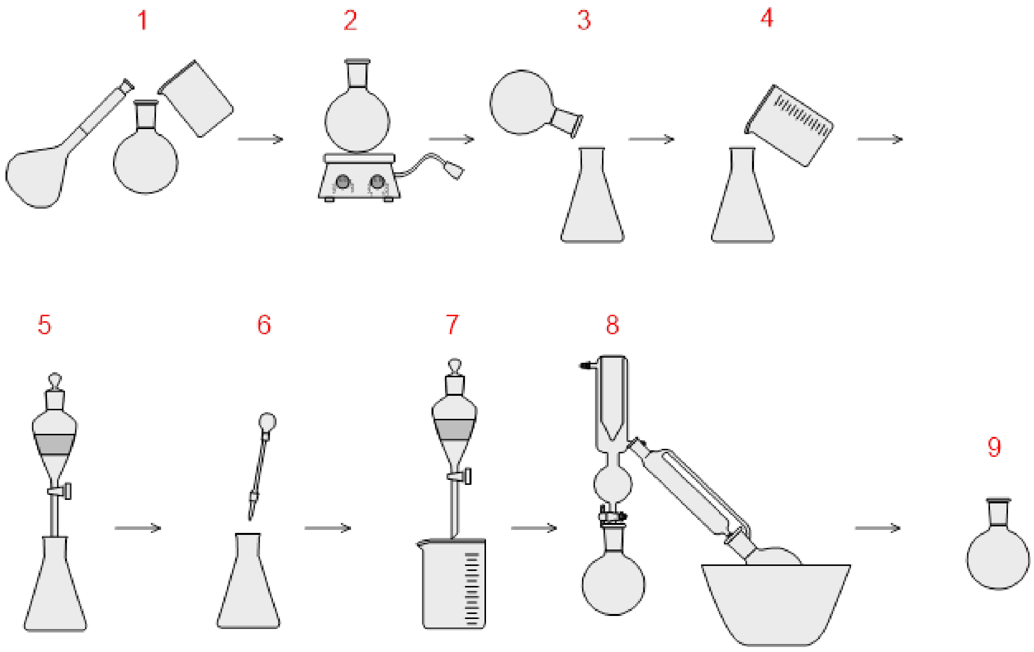

Figure S1: General Procedure for Coconut Oil Hydrolysis

Caption: 1 - 30 mL of ethanolic solution and 5.0 g of oil were mixed in a round-bottom flask; 2 - round-bottom flask with the reaction medium under stirring and heating; 3 - the reaction was transferred to a beaker; 4 - 30 mL of water were added; 5 - liquid-liquid separation with hexane; 6 - acidified alcoholic phase; 7 - fatty acids were recovered by hexane extraction, followed by washing with water until pH neutrality; 8 - rotary evaporation to separate fatty acids from the hexane fraction; 9 - fatty acids obtained.

2. General Procedure for Electrodescarboxylation

For the electrolysis reactions, graphite electrodes with dimensions of 10 mm × 50 mm × 3 mm were used (Figure S2). When placed in the reaction phase, the submerged surface was 10 mm × 20 mm × 3 mm. The experiments at constant potential were conducted using a DC bench power supply (Wanptek, KPS305D) in an electrochemical cell composed of a glass tube (9 cm in length, Ø = 2 cm), connected by crocodile clips, electrodes separated by a distance of 8 mm, supported by copper filaments, and fixed using a polyethylene piece. Magnetic stir bar (5 × 10 mm), magnetic stirrer, universal stand, clamp, and three-finger grip were utilized. Figures S2 and S3 illustrate the assembly of the electrochemical cell and the electrochemical setup. The graphite electrodes, commercially obtained, underwent a surface cleaning procedure using 600-grit wet sandpaper. Subsequently, they were immersed in an ultrasonic bath for 15 minutes in sulfuric acid (ISOFAR, ≥ 98%) and then placed in distilled water for 10 minutes.

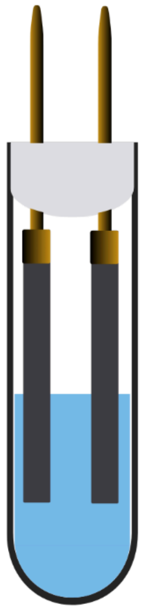

Figure S2: Illustrative scheme of the electrochemical cell used for the electrocarboxylation of fatty acids.

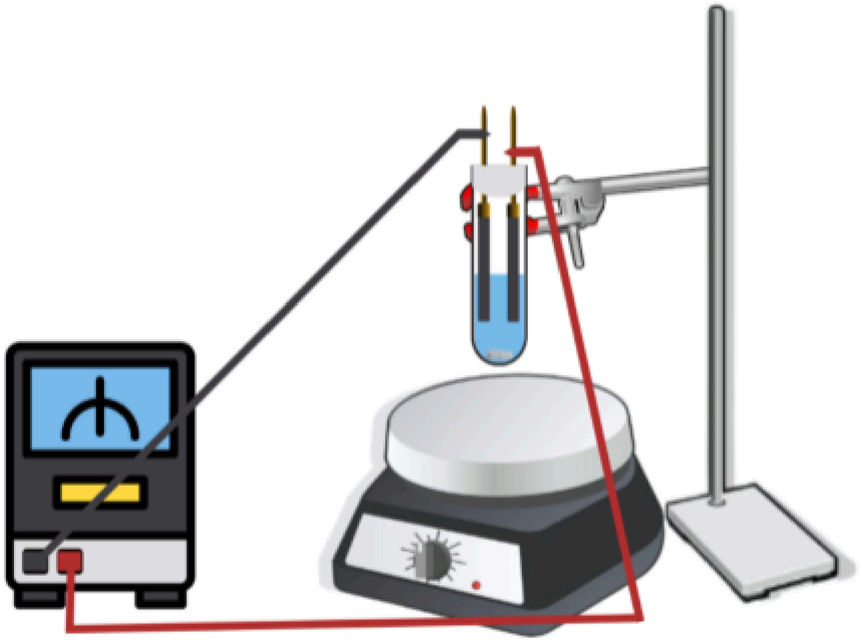

Figure S3: Illustrative scheme of the electrochemical setup used for the electrocarboxylation of fatty acids.

The methodology for analyzing the influence of parameters in the electrocarboxylation of fatty acids was guided by the yield of relative distribution through GC-MS. The variables solvent, electrolytes (bases), and potential were analyzed with the best relative distributions.

The reactions were prepared according to the following procedure: In an electrochemical cell, clean and dried in an oven, equipped with a magnetic stirring bar, fatty acids derived from coconut oil (177 mg, 0.8 mmol, 1 equiv) were added, along with 10 mL of solvent and the base (0.8 mmol, 1 equiv). The reaction was kept at room temperature under stirring until the complete dissolution of the base. Subsequently, the cell was sealed with a polyethylene holder containing copper filaments connected to graphite electrodes, which were connected by crocodile clips to a DC bench power supply, with a constant potential applied for 3 hours at room temperature (Figure S3).

For the analysis of the reaction products of fatty acids, a 1 mL aliquot of the reaction was taken and placed in a borosilicate flask, along with the addition of 1.0 mL of 1.0 mol L^{-1} hydrochloric acid solution and 2 mL of hexane. After phase separation, a 200 µL aliquot of the hexane fraction was taken and transferred to another flask. Then, 1.8 mL of UV/HPLC-grade n-hexane was added. In this organic phase, anhydrous sodium sulfate was added, acting as a drying agent, and it was then sent for qualitative analysis by GC-MS. The chromatograms were used to determine the relative distribution fractions of each product, as well as their conversion.

3. Gas Chromatography Coupled with Mass Spectrometry (GC-MS)

The relative yields were obtained using a Shimadzu gas chromatograph (GC-2010) coupled to a mass spectrometry detector (GCMS-QP2020). The equipment features an RTX-5MS column packed with 5% diphenyl and 95% dimethylpolysiloxane, with dimensions of 30 m in length, 0.25 mm inner diameter, and 0.25 μm film thickness. Samples were prepared in UV/HPLC-grade hexane, and the injection was performed at a temperature of 230°C with an injection flow rate of 1 mL/min, using highly pure helium as the carrier gas. The program used for chromatographic separation employed an initial temperature of 40°C maintained for 4 minutes, followed by a heating ramp at a rate of 4°C/min up to 180°C, holding this temperature for 5 minutes. Then, the temperature was increased at a rate of 7°C/min up to 270°C (maintained for 3 minutes).

4. Materials

4.1 Solvents

The solvents used in the procedures were: ethyl acetate, acetone, acetonitrile, isopropyl alcohol, anisole, chloroform, dimethylacetamide, dimethylformamide, dimethyl sulfoxide, dioxane, ethanol, di-isopropyl ether, ethyl ether, hexane, methanol, methylpyrrolidine, tetrahydrofuran, toluene. All of them were obtained from commercial sources (Dinâmica, Sigma-Aldrich, Synth, Vetec, and ACS científica) and used without any prior treatment or purification.

4.2 Reagents

The reagents used in the procedures were: 37% hydrochloric acid, sodium bicarbonate, diethylamine, potassium hydroxide, sodium hydroxide, N,N-diisopropylethylamine (DIPEA), pyridine, sodium sulfate, triethylamine. These items were commercially obtained (Dinâmica, Sigma-Aldrich, and Synth).

5. References

[1] Salimon J, Abdullah BM, Salih N. Hydrolysis optimization and characterization study of preparing fatty acids from *Jatropha curcas* seed oil. *Chem. Cent. J.* 2011;5(1):1–9. https://doi.org/10.1186/1752-153X-5-67
